# The construction and analysis of a ferroptosis-related gene prognostic signature for pancreatic cancer

**DOI:** 10.18632/aging.202801

**Published:** 2021-04-04

**Authors:** Peicheng Jiang, Feng Yang, Caifeng Zou, Tianyuan Bao, Mengmeng Wu, Dongqin Yang, Shurui Bu

**Affiliations:** 1 Department of Gastroenterology, Fudan University Jinshan Hospital, Shanghai, China; 2 Department of Pancreatic Surgery, Fudan University Huashan Hospital, Shanghai, China; 3 Department of Digestive Diseases, Fudan University Huashan Hospital, Shanghai, China

**Keywords:** ferroptosis, pancreatic cancer, prognostic signature, survival analysis, immune checkpoint blockade

## Abstract

Ferroptosis is a regulated cell death nexus linking metabolism, redox biology and diseases including cancer. The aim of the present study was to identify a ferroptosis-related gene prognostic signature for pancreatic cancer (PCa) by systematic analysis of transcriptional profiles from Cancer Genome Atlas (TCGA) and Genotype-Tissue Expression (GTEx). Altogether 14 ferroptosis-relevant genes with potential prognostic values were identified, based on which a risk score formula was constructed. According to the risk scores, we classified the patients into a high- and a low-risk score group. It was verified in Gene Expression Omnibus (GEO) and ICGC (International Cancer Genome Consortium) datasets. The Kaplan-Meier survival curves demonstrated that patients with lower risk scores had significantly favorable overall survival (OS) (P < 0.0001). The area under the receiver operating curve (ROC) for 12, 18 and 24 months was about 0.8 in all patients. The result of immune status analysis revealed that the signature significantly associated with the immune infiltration and immune checkpoint blockade (ICB) proteins. In addition, we used quantitative real time PCR (q-rtPCR) and Human Protein Atlas (HPA) to validate the expression of the key genes. Collectively, the signature is valuable for survival prediction of PCa patients. As the signature also has relevance with the immune characteristics, it may help improve the efficacy of personalized immunotherapy.

## INTRODUCTION

Pancreatic cancer (PCa) is a malignant tumor with high mortality [1]. The five-year survival rate of PCa patients who received surgical resection is only 10-25% [2]. About 80-85% patients with PCa patients presented with unresectable status or metastasis at the time of diagnosis [3], for whom the systemic chemotherapy is the main treatment at present but the survival outcome is often unsatisfactory [4].

Immunotherapy is an innovative treatment strategy in oncology, which offers the promise in the treatment of cancer and has achieved satisfactory outcomes in various malignancies as expected. Targeting immune checkpoint molecules with immune checkpoint inhibitors has opened up a new vista in cancer treatment [5–7]. In 2017, the US Food and Drug Administration (FDA) approved the PD-1 blocker pembrolizumab for tumor patients who are identified as deficient mismatch repair (dMMR) or high microsatellite instability (MSI-H), including PCa [8]. However, this benefit of immunotherapy has not lived up to expectations in most PCa patients because of the complex, highly immunosuppressive microenvironment [9]. Even so, it does not mean a desperate plight of immune therapy in pancreatic cancer. Balachandran et al [10] observed long-term survival of PCa patients and identified unique neoantigens responding to T cells that prompted the specific immune microenvironment. A study on combination immunotherapy also brings hope to pancreatic cancer patients by prolonging their overall survival (OS) [11].

The concept of ferroptosis was first proposed by Stockwell's group in 2012 based on their finding of catastrophic accumulation of lipid reactive oxygen species (ROS) and abnormal iron metabolism [12]. Ferroptosis is a newly discovered form of programmed cell death which is distinct from apoptosis, necroptosis, and autophagy in cell morphology and function [13]. Some recent studies have implicated that ferroptosis is involved in multiple physiological and pathological processes of many diseases including cancer [14, 15]. Other studies also demonstrated that ferroptosis is an important mechanism by which some drugs promoted the death of various PCa cell lines [16, 17]. Michael A et al [18] recently reported their finding in the journal Science that cysteine depletion induced tumor-selective ferroptosis and inhibited cell growth in genetically engineered PCa mice. As one of the most impactful classes of anti-cancer therapies, immune checkpoint blockade (ICB) therapies have also drawn increasing attention of researchers engaged in ferroptosis. Wang et al [19] reported that CD8+ T activation cells could enhance ferroptosis-specific lipid peroxidation in tumor cells, thus increasing ferroptosis and contributing to the anti-tumor efficacy of immunotherapy.

Based on the above findings, we speculated that ferroptosis-related genes (FRGs) may have a prognostic value for PCa patients. By integrating a series of systematic analyses to multiple datasets, we constructed and validated a robust and practical molecular signature of FRGs for survival of PCa patients, finding that the signature constructed in this study was associated with tumor immunity. All in all, we have built a reliable FRGs prognostic signature and uncovered a potential ICB biomarker for PCa.

## RESULTS

### Identification and functional enrichment analysis of DEGs

**Figure 1:**
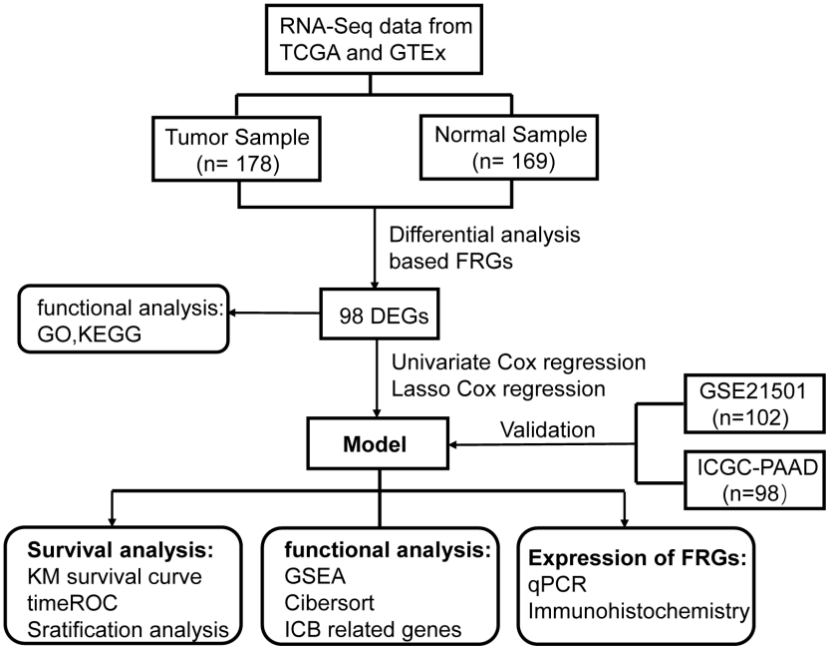
**Flowchart of overall study design.**

**Figure 2:**
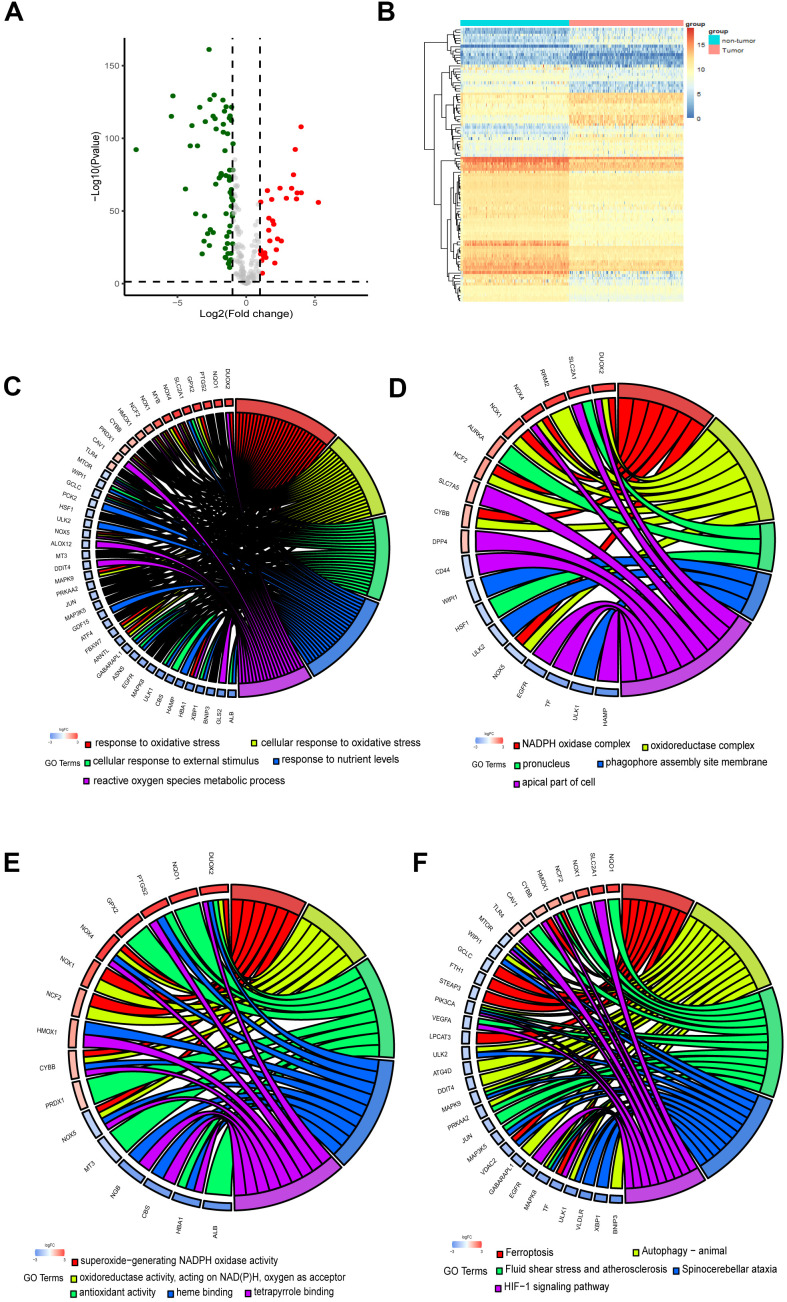
**Identification and functional enrichment analysis of the ferroptosis-related DEGs.** (**A**) Volcano plot of DEGs. Red dots represented up-regulated genes while green dots represented down-regulated genes, and black dots represented no differences gene. (**B**) Heatmap of DEGs to visualize the expression levels of genes. (**C**–**F**) Chord plot depicting the relationship between DEGs and GO in terms of the biological process, cellular component, molecular function and KEGG pathways.

The flow chart of the present study is shown in Figure 1. The data of The Cancer Genome Atlas (TCGA) and Genotype-Tissue Expression (GTEx) was downloaded from UCSC Xena Database. After screening and classification, RNA-seq data and clinical information of 178 pancreatic cancer samples and 169 normal samples were left for subsequent analyses. The clinical characteristics of the included patients are presented in Supplementary Table 1. Based on the coding FRGs from the publications, we detected the expression of FRGs by differential analysis and found that 30 FRGs were up-regulated and 68 FRGs were down-regulated (P<0.05, |log2FC| >1.0) (Supplementary Table 2). The distribution of these differently expressed FRGs is displayed in Figure 2A, 2B. These 98 differentially expressed FRGs were further analyzed by GO and KEGG pathway to explore their functions.

As the chord plots shown (Figure 2C–2E), response to oxidative stress, superoxide−generating NADPH oxidase activity, reactive oxygen species metabolic process and oxidoreductase complex were the most enriched biological processes. The involvement of these biological processes and complexes in ferroptosis has been reported [20, 21]. Besides, the DEGs were significantly enriched in response to nutrient levels. As KEGG pathway analysis revealed, the DEGs participated in autophagy, HIF−1 signaling pathway and Foxo signaling pathway (Figure 2F).

### Establishment of the ferroptosis-related prognostic signature in pancreatic cancer

**Figure 3:**
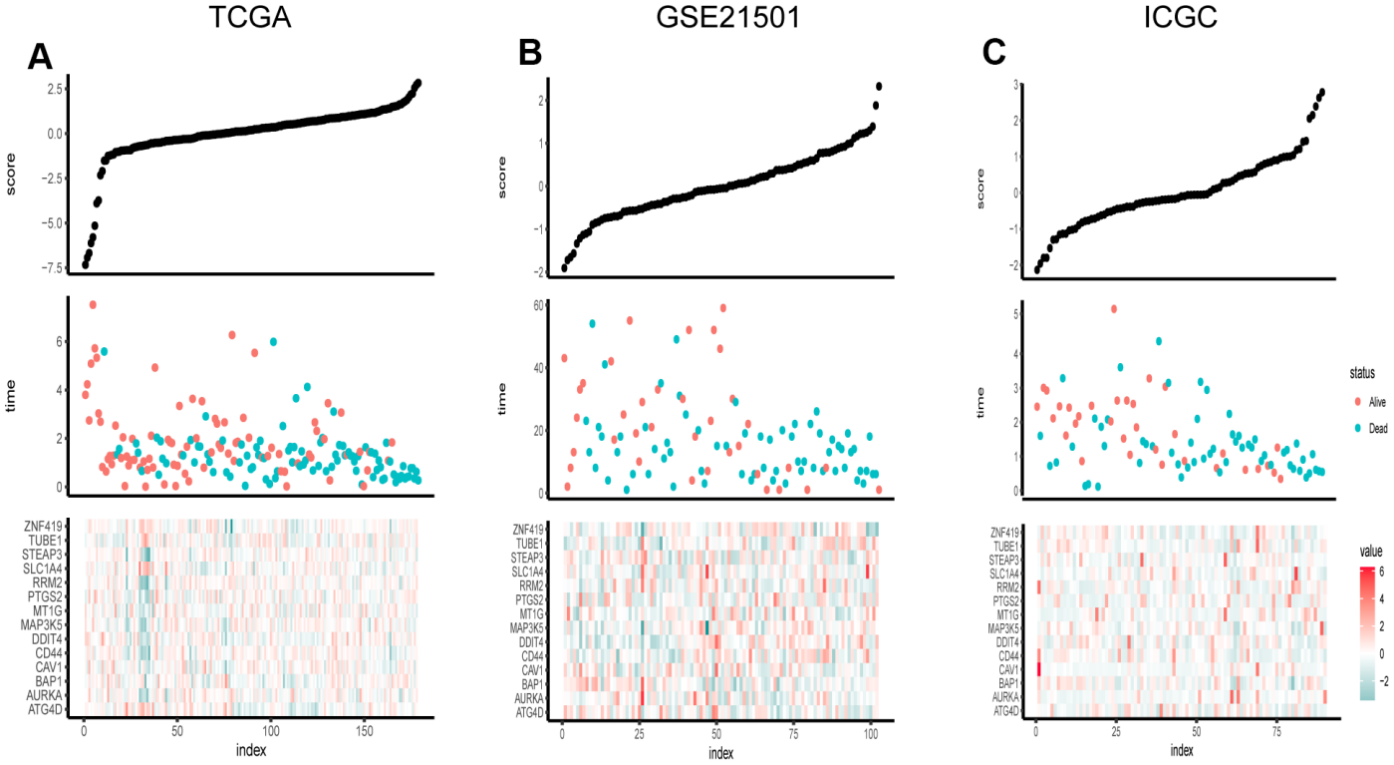
Risk score distribution, survival overview and heatmap of key genes in the TCGA (**A**), GSE21501 (**B**), and ICGC (**C**). The heatmaps were applied to visualize the expression levels of key genes in every sample.

Univariate Cox regression analysis and the least absolute shrinkage and selection operator (LASSO) Cox regression model were applied to evaluate these differentially expressed FRGs in the training set for the sake of finding key genes most associated with the prognosis of pancreatic cancer (Figure 3A, 3B). As a result, 14 prognosis-related key FRGs were identified and integrated to construct a prognostic signature for PCa.

For further exploring the significance of risk score, 178 patients were classified into two groups on the basis of the median FRG score value. The distribution of risk scores is presented in Figure 3C. The result showed that the higher the risk score, the more densely the state of death was distributed, indicating that the FRG score was accurate reliable in predicting the prognosis and survival of PCa patients.

**Figure 4:**
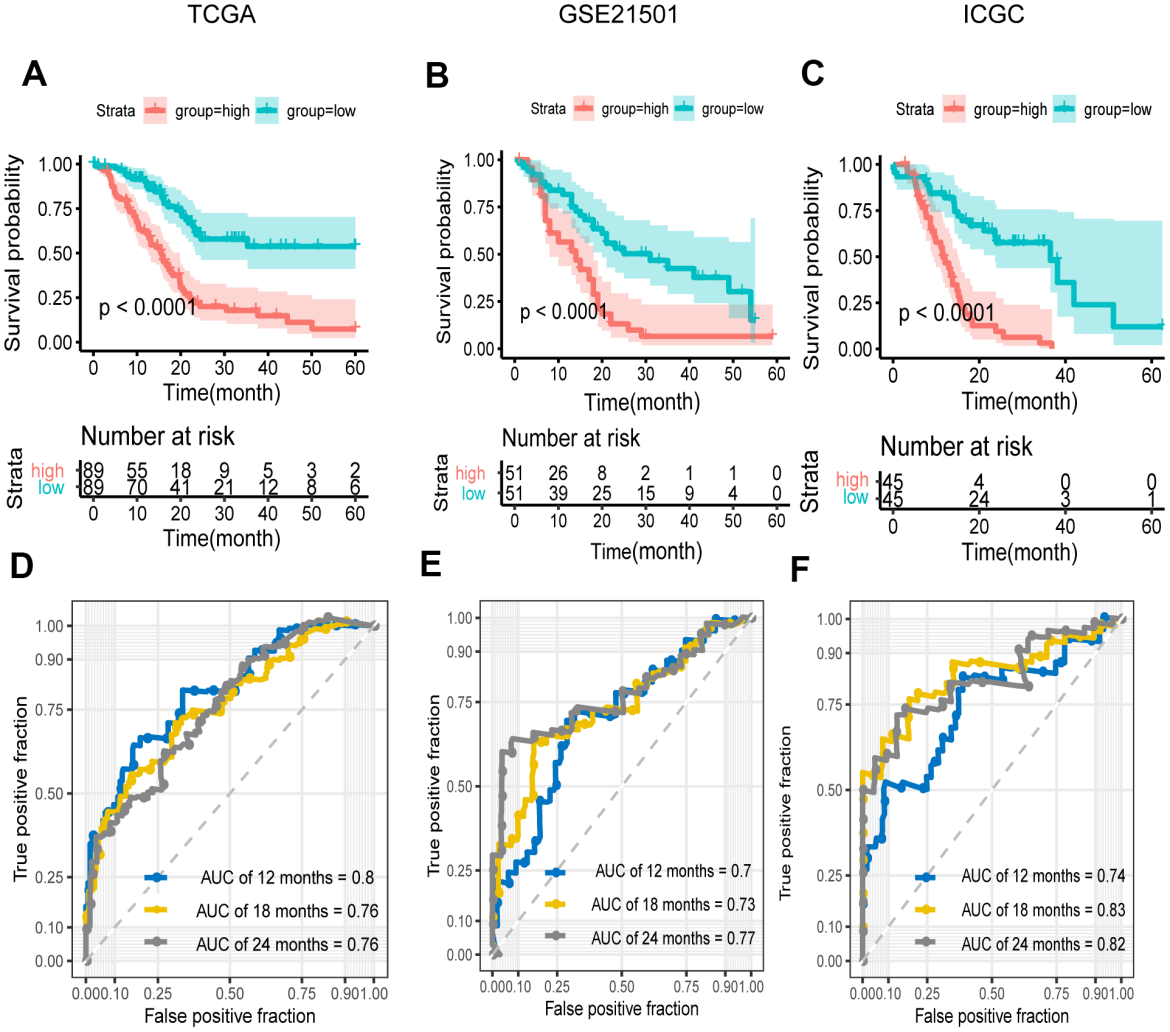
**Survival and ROC analysis in training and validation datasets.** (**A**–**C**) Kaplan–Meier overall survival curves for patients in high- and low-risk groups of the TCGA (**A**), GEO (**B**), and ICGC (**C**) datasets. Hazard ratios (HRs) and 95% CIs are for risk group. P values were calculated with the log-rank test. (**D**–**F**) Time-dependent ROC curves at 12, 18, 24 months for patients in the TCGA (**D**), GSE21501 (**E**), and ICGC (**F**) datasets to evaluate the prediction efficiency of the prognostic signature.

Kaplan–Meier curve and time-dependent ROC were generated to evaluate the prognostic capability of the signature. We applied the same procedures to the data of GSE21501 and ICGC-PACA. The number of cases in the two sets was 102 and 90 respectively. The results presented an satisfactory ability of the signature for survival prediction. The Kaplan-Meier survival curves showed that OS in low-risk group was significantly longer than that in high-risk group (P < 0.0001) (Figure 4A–4C). The area under the curve (AUCs) for 12, 18 and 24 months were 0.8, 0.76 and 0.76 in the training set respectively, and the value for two cohorts were 0.7, 0.73 and 0.77 vs. 0.74, 0.83 and 0.82 respectively (Figure 4D–4F), while these for the TNM stage and AJCC stage were 0.59, 0.62 and 0.68 vs. 0.49, 0.55 and 0.65 (Supplementary Figure 1). These results demonstrated that our signature in predicting prognosis was superior to the conventional classification.

### Applicability of the FRG signature as an independent prognostic indicator

To explore whether the prognostic model was independent of conventional clinical factors, we used univariate cox regression to analyze the PCa patients from the TCGA cohort. The result showed that our risk score, T stage and N stage were all statistically significant in predicting prognosis. By incorporating the three variables, we further performed multivariate cox regression analysis and verified that our prognosis signature was a significant and independent factor (P=2.25e-10) (Table 1).

**Table 1:** Univariate and multivariate cox regression models of the FRG signature in predicting prognosis.

**Variables**	**Univariate cox model**			**Multivariate cox model**	
	**HR**	**P value**		**HR**	**P value**
age	1.42236662	0.123471			
gender	0.82345753	0.350272			
grade	1.51438806	0.059922			
AJCC_stage	0.19668764	0.397011			
TumorT (3-4 vs. 1-2)*	2.32464502	0.009336		-0.03486	0.917
TumorN (1 vs. 0)*	2.20904129	0.001905		0.30990	0.244
score	2.71828183	1.78E-11		0.96209	2.25e-10

*tumor node metastasis classification. T, tumor; N, node.

**Figure 5:**
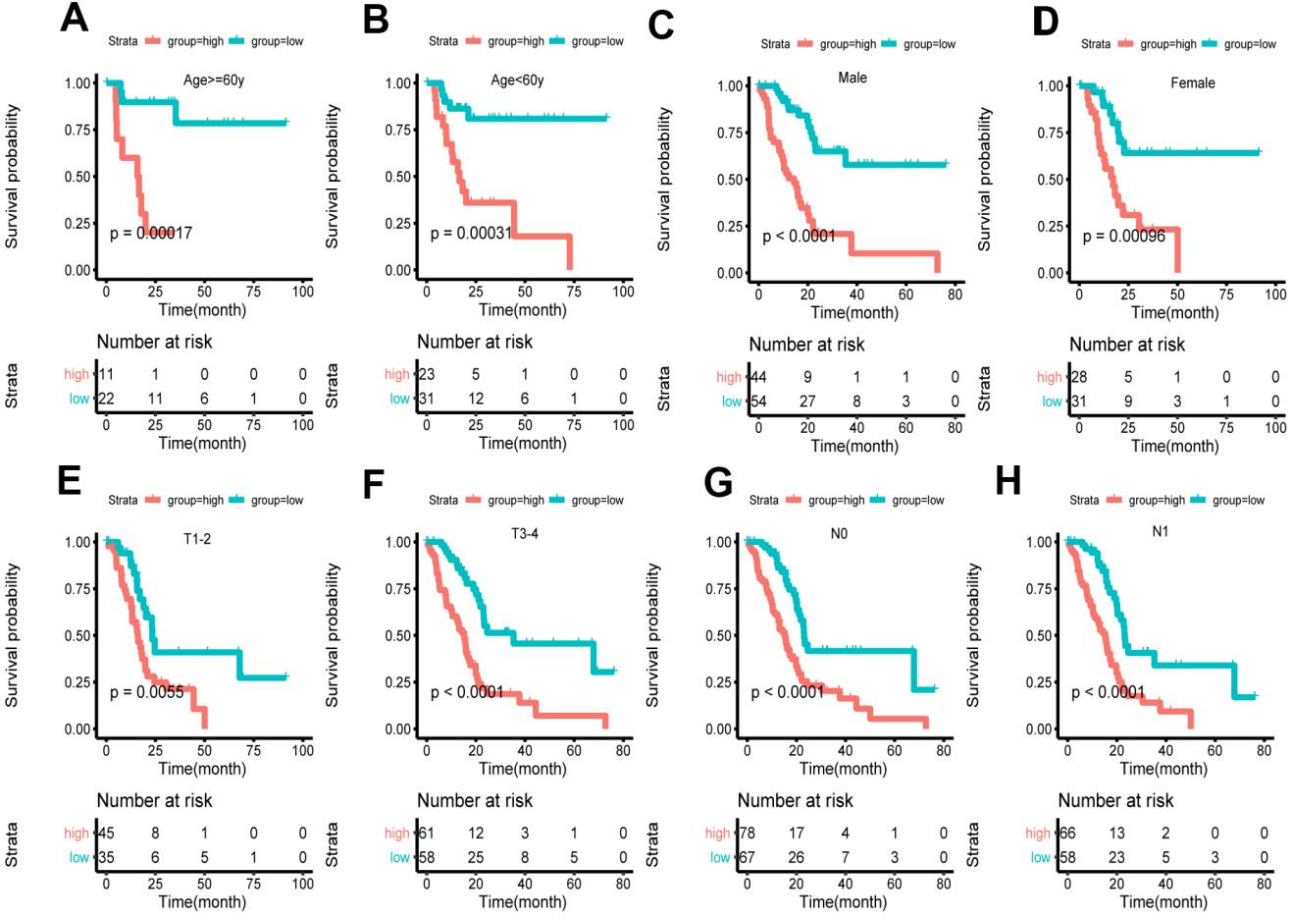
**Survival of the FRG signature in patients stratified by gender, age and tumor stage.** (**A**, **B**) The difference in OS between high- and low-risk group stratified by age. (**C**, **D**) The difference in OS between high- and low-risk group stratified by gender. (**E**, **F**) The difference in OS between high- and low-risk group stratified by T stage. (**G**, **H**) The difference in OS between high- and low-risk group stratified by N stage. (**E**, **F**) According to tumor node metastasis classification. T, tumor; N, node.

Based on clinical character including age, gender and tumor stage, we stratified the whole cohort from TCGA to ensure the applicability of this signature. The result of stratification analysis showed that disease-free survival (DFS) in high-risk group patients was significantly shorter than that in low-risk group patients in all age groups (P < 0.05) (Figure 5A, 5B). Stratification analysis of gender and tumor stage presented the same tendency (P < 0.05) (Figure 5C–5H). Taken together, the ferroptosis-related signature was practicable and reliable for predicting survival in multiple strata of patients.

### Correlation of the FRG signature with tumor immunity associated characteristics

**Figure 6:**
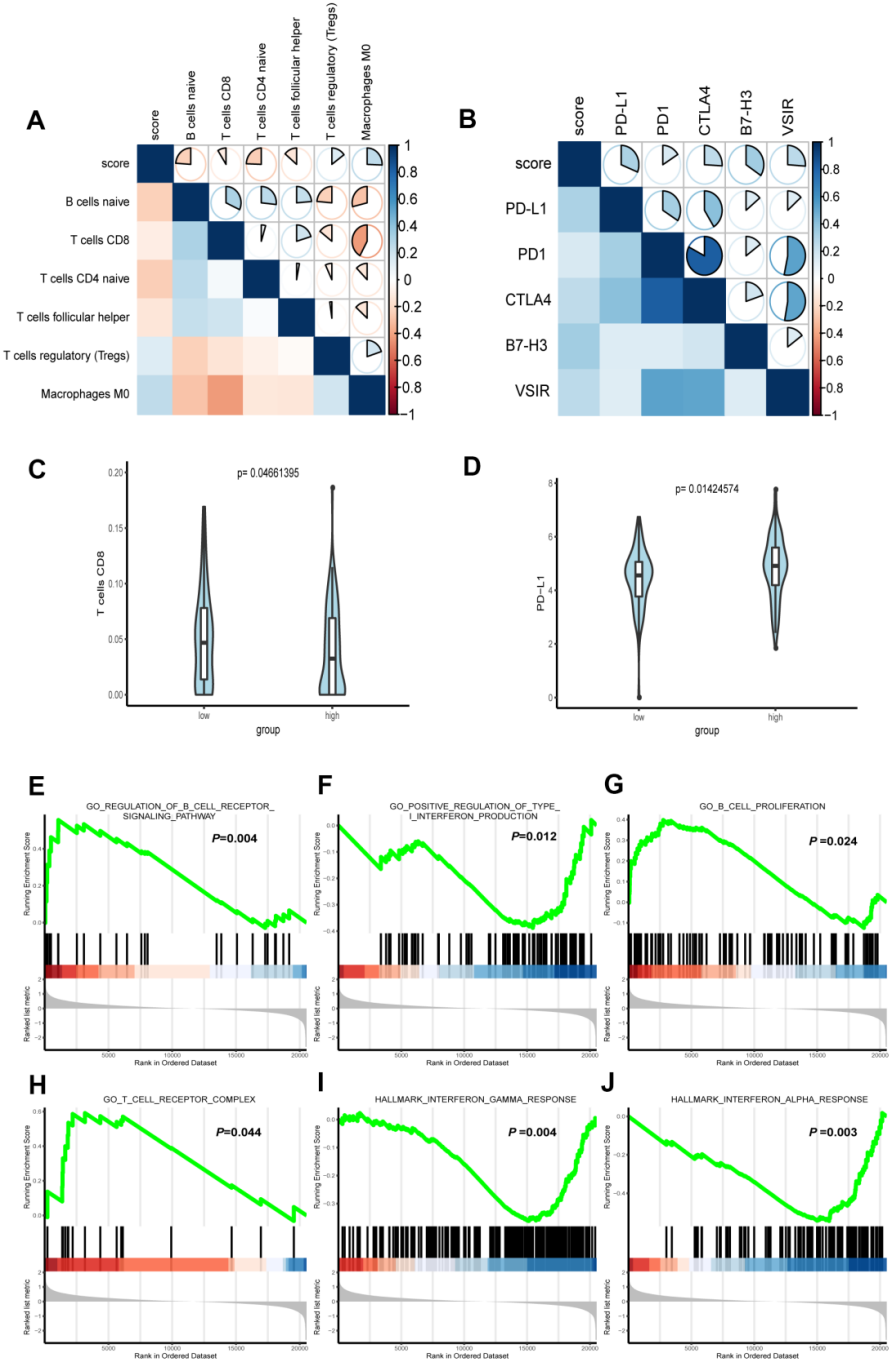
**Correlation between immune characteristics with the FRG signature.** (**A**) Spearman correlation analysis was conducted to determine the correlation of immune cells with risk score of the signature. (**B**) Spearman correlation analysis was conducted to determine the correlation of immune checkpoint inhibitors PD-1, PD-L1, CTLA-4, B7H3, and VSIR with risk score of the signature. (**C**) CD8+ T cell infiltration in high- and low-risk groups. (**D**) The expression level of PD-L1 in high- and low-risk groups. (**E**–**J**) Representative enriched pathways in immune character associated with FRG signature by GSEA analysis.

To acquire the relative proportions of 22 immune cell subsets of LGG, we applied CIBERSORT algorithm and performed correlation analysis to uncover the relevance of the score and immune cells. As shown in Figure 6A, naive B cells, CD8+ T cells, T naïve CD4 cells and follicular helper T cells were positively correlated with the score. On the contrary, Treg cells, Macrophages M0 were negatively correlated with the score.

PD-1, PD-L1 and CTLA-4 are known biomarkers for tumor immunity and have won general recognition in immunotherapy [22]. Both B7-H3 and VSIR are immunoregulatory proteins, which are overexpressed in various cancers and associated with poor prognosis [23]. The role of B7-H3 in oncogenesis and progress indicates its potential as a biomarker and immunotherapy target [24]. VISTA, which has high similarities with PD-1 [25], interferes with the antigen presentation to suppress T cell responses [26]. To explore the link of our formula and ICB immunotherapy-related signatures, we conducted Spearman correlation to analyze the RNA-seq data from TCGA. The result demonstrated that PD-L1, PD1, CTLA4, B7-H3 and VSIR all played a role in regulating tumor immunity and were inversely correlated with the score (Figure 6B).

In addition, we conducted further analysis to test its significance for immunity character. The Wilcoxon rank-sum test revealed that CD8+ T cell and PD-L1, as the immune cell and ICB related molecules that aroused the clinical concern most were significantly higher than those in low-risk group (Figure 6C, 6D).

We further used gene set enrichment analysis (GSEA) to analyze the difference of enriched gene sets. Setting P <0.05 as the cutoff value, we found that multiple immunity-associated pathways were involved (Figure 6E–6J), indicating that lower risk scores were associated with better antitumor immunity, including positive regulation of B cell receptor signaling pathway and higher B cell proliferation as well as T cell receptor complex. Yet, a higher risk score was associated with the impaired production of type I interferon in antitumor immunity, as well as interferon α and γ response.

### Expression levels of key genes in the clinical samples

**Figure 7:**
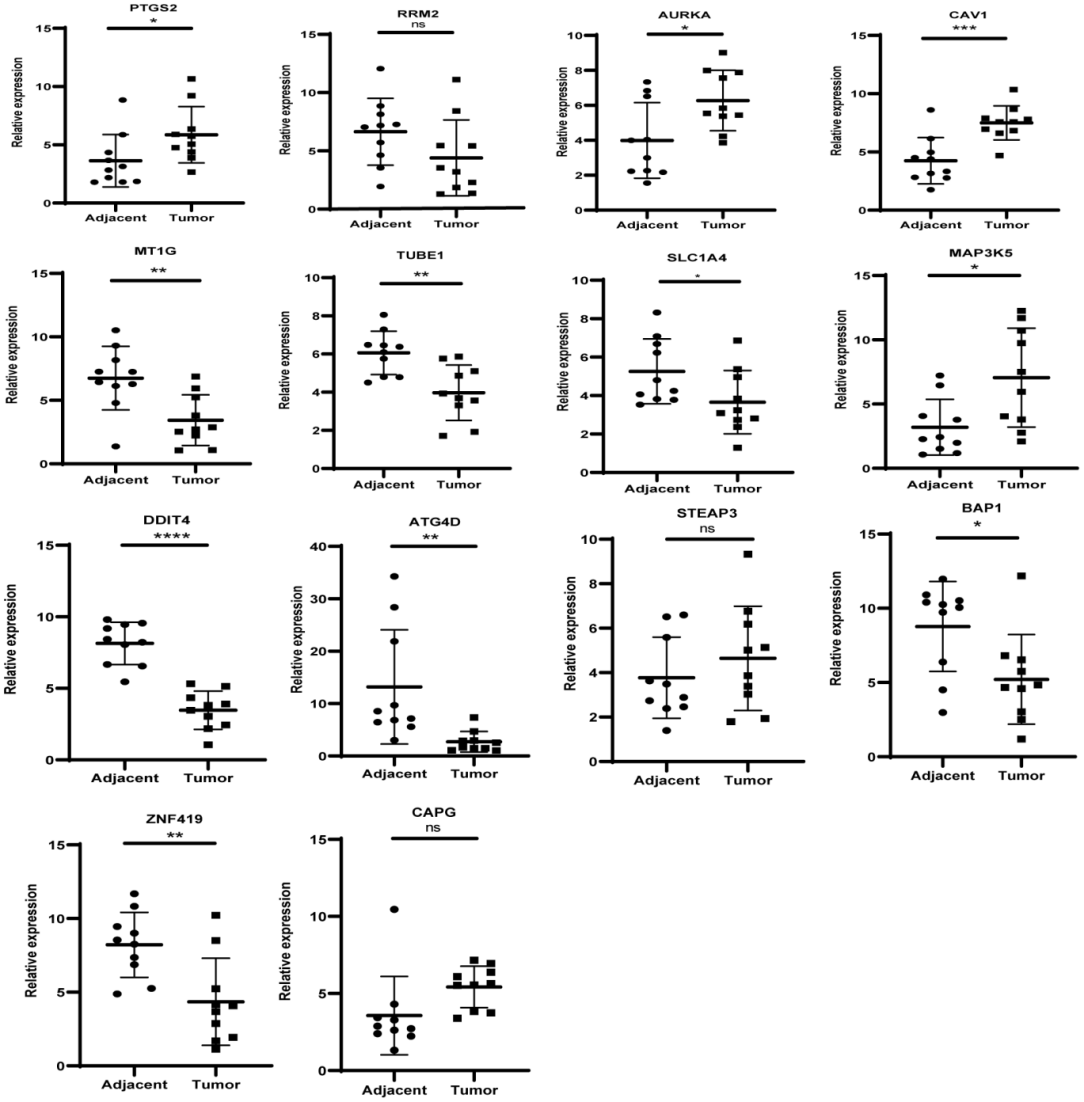
**The mRNA expression level of the key genes in the clinical samples.**

**Figure 8:**
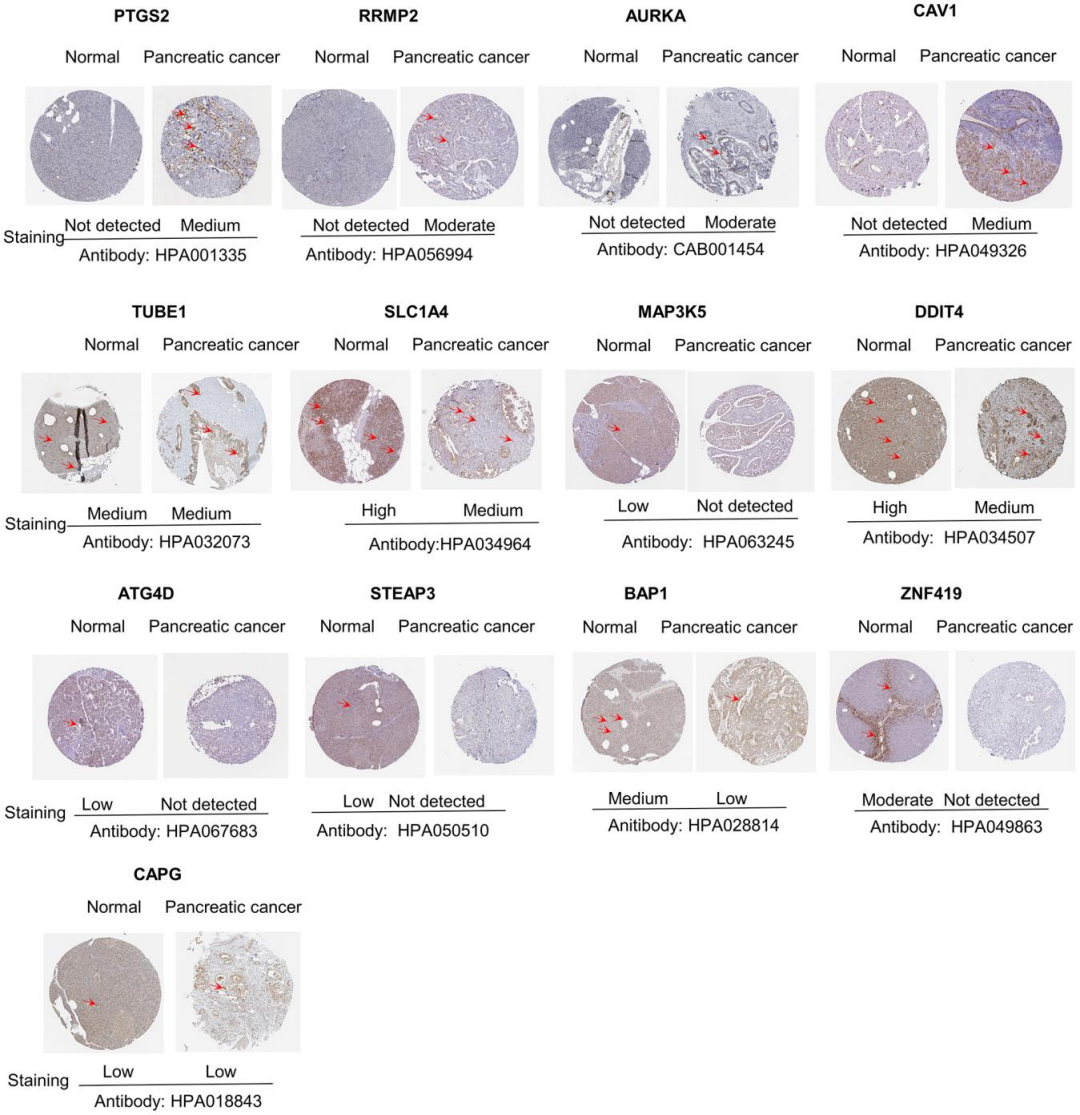
**Differences in protein expression of the key genes in pancreatic cancer tumor tissue and normal tissue from Human Protein Atlas immunohistochemistry.**

The levels of the 14 genes in the PCa and paired adjacent normal tissues were compared to explore the clinical significance of the signature. The clinicopathological parameters of patients were presented in Supplementary Table 4. The result of q-rtPCR showed that the mRNA expression levels of PTGS2, RRM2, AURKA, CAV1, MAP3K5, STEAPS are higher in tumor samples and lower in normal tissue samples and the others had the reverse tendency (Figure 7). To investigate the protein expression of key FRGs, we studied the immunohistochemistry results using the Human Protein Atlas database in normal pancreas tissue and tumor tissue. Except for MT1G, the other proteins available were nearly consistent with the results of TCGA database as well as qRT-PCR (Figure 8).

Considering the coefficient of the formula, we reasoned that upregulation of PTGS2 and downregulation of *MT1G, TUBE1 and ATG4D* might have comprehensive effect on increasing the risk of tumorigenesis and poor prognosis.

## DISCUSSION

Pancreatic cancer is a malignant tumor with high morbidity and mortality, imposing a high socioeconomic burden. Although tremendous advances have been made in cancer treatment and translational medicine, surgical resection remains the main treatment for PCa. As most PCa patients have lost the chance of surgery and the outcome of chemotherapy remains unsatisfactory, the prognosis of PCa is relatively poor. the emergence of immunotherapy in recent years has brought renaissance in oncology therapeutics. Little infiltration of effector T cells and few immunogenic antigens contribute to low response to immunotherapy in PCa [27, 28]. Nevertheless, part of PCa patients with relatively good immune conditions can still achieve a relatively long OS [29, 30], suggesting that immunotherapy has a promising prospect in the treatment of PCa. Ferroptosis is a recently discovered type of cell death and plays an important regulatory role in various cancer types including PCa [31–33]. Gemcitabine is the standard therapy for PCa. ARF6 can mitigate gemcitabine resistance by conferring PCa cells the sensitivity to RSL3-induced ferroptosis [34]. Studies have demonstrated that multiple chemical extracts can promote the death of pancreatic cancer cells mainly through the induction of ferroptosis [17, 35, 36]. Ferroptosis induced by SLC7A11 deletion has be verified to slow down the growth of the xenograft derived from PCa [37]. The research conducted by DeNicola’s team may partly explain the basis of the link between ferroptosis and PCa. ROS is a product of mutant-KRAS signaling, which is involved in various metabolic processes and may thereby be a pro-tumor factor. Of note, over 90% pancreatic adenocarcinoma patients burden mutations in KRAS. PCa cells take advantage of cysteine-derived metabolites such as glutathione (GSH) to counteract the increased ROS, the accumulation of which is known to trigger ferroptosis [38]. The existing research results tempted us to consider the connection between ferroptosis and prognosis of PCa. Based on TCGA, we established a robust FRG and verified it in GSE21501 and ICGC-PACA datasets. The result showed that it had a high AUC value.

The role of ferroptosis in immunotherapy has aroused much attention and interest in recent years. In one study of immunotherapy-associated cytokines, the researchers observed that the inducers of ferroptosis had impact on the differentiation of melanoma cells and affected the anti-tumor efficacy of immunotherapy [39]. Some physiological processes induced by ferroptosis could to some extent activate innate immunity [40]. The engulfment of ferroptotic cancer cells by macrophages indicated that ferroptotic cells may attract antigen-presenting cells (APCs), thereby increasing the anticancer immunity [41, 42]. Another study demonstrated that the release of RAGE triggered by ferroptotic cells was necessary for HMGB1-mediated tumor necrosis factor (TNF) production in macrophages, suggesting that ferroptotic cancer cells could be immunogenic in nature [43]. Accumulation of lethal lipid peroxides is one of the fundamental biological processes of ferroptosis, knowing that tumor immunity is modulated by the interaction between ferroptosis and lipid metabolism [44]. At the same time, immune cells also have impact on ferroptosis. It is reported that oxidized phospholipids formed in immune cells can promote ferroptosis [45, 46]. High expression of PTGS in ferroptotic tumor cells can impair the anti-tumor effect of immune cells [47, 48]. To explore whether the FRG signature had a value in personalized immunotherapy for PCa, we performed GSEA for further functional analysis and found that the score was negatively correlated with the response of antitumor immunity.

Given the above situation, we intended to unearth more information about the immunological characteristics of the FRG signature. Cibersort and Spearman correlation analyses were performed to define the character of immune cell infiltration and the expression of immunotherapy-related molecules. It was found that immune cells including antitumor cells such as T cell CD4+ naïve and CD8+ T cells were inversely correlated with the score, while immunosuppressive cells such as Treg cells were positively associated with the score, which may explain the poor prognosis of the patients in high risk group. The high infiltration of CD4+ T cells may mean longer OS for PCa patients [49]. Multiple pre-clinical cancer models and the responses of patients have proved that the efficacy of checkpoint blockade immunotherapy is strongly associated with the number and status of CD8+ T cells [50–52]. The results of some studies have demonstrated that the immunosuppressive effect of Treg plays a role in the pathogenesis of PCa [53, 54]. The analysis of immunotherapy-related genes showed that immunosuppressive molecules such as PD-L1, CTLA4 were positively correlated with the score, indicating the potential of the high risk group in ICB therapies.

It is well established that CD8 cell infiltration to the tumor microenvironment is linked with a favorable prognostic outcome. PD-1 and its ligand constitute an essential inhibitory mechanism causing T cell exhaustion, which is inclined to the immunotolerant environment in tumors. That's the main reason why PD-L1 has drawn increasing attention of researchers concerned [55]. PCa patients with high PD-L1 expression were found to have an immunosuppressive tumor microenvironment, in whom the cytotoxic effect of activated T-cells was inhibited [56]. These findings are consistent with the results of CD8+ T distribution and PD-L1 level in our two groups, which to some extent demonstrates the reliability of our model in the immunological character.

In summary, we identified 14 ferroptosis-related prognostic genes in PCa by comprehensive mining of the transcriptional profiles. The signature described herein performed well in risk stratification in the training cohort and the two independent cohorts. In addition, further analysis revealed that its link to the immunological character facilitated evaluating the personalized response to ICB immunotherapy. It may serve as a classifier for clinical decision-making regarding individualized prognosis and the response to anti-tumor immunotherapy.

## MATERIALS AND METHODS

### Ethical statement

All patients signed the informed consent forms before initiation of the study. Collection of the clinical specimens was in accordance with the national and international guidelines involving Declaration of Helsinki.

### Collection of the clinical samples

Altogether 10 pairs of PCa and adjacent non-tumor tissues were collected from the patients who received surgical resection in Huashan Hospital affiliated to Fudan University (Shanghai, China) from May to October 2019. All these tissue samples were frozen in liquid nitrogen as soon as they were isolated and then stored at –80° C for analysis.

### Patient recruitment and data preparation

For the transcriptional profiles in the training cohort, Cancer Genome Atlas (TCGA) data and Genotype-Tissue Expression (GTEx) data of pancreatic cancer were obtained from UCSC Xena Database (http://xena.ucsc.edu/), and the transcriptome data provided by the database were normalized with the log2(x+1) transformation. The data acquired from GTEx are all from healthy donators. In order to further validate the results from Gene Expression Omnibus (GEO), we acquired the data of GSE21501 dataset from GEO (http://www.ncbi.nlm.nih.gov/geo) after systematical screening. The criteria were as follows: the information of samples was relatively complete and substantial. The RNA-seq data and clinical information of ICGC- PACA-AU from the ICGC portal were downloaded from the official website (https://dcc.icgc.org). The normalized read count values were used. Cases with vague or absent clinical outcome information were excluded.

### Identification of key prognostic genes and establishment of the model

FerrDb database (http://www.zhounan.org/ferrdb/index.html) is the world’s first database for ferroptosis, providing an updated database for regulators and markers as well ferroptosis-disease associations. Altogether 256 coding FRGs were acquired from the website. Subsequently, we performed a differential expression analysis on the data from TCGA and GTEx [57] and identified 98 DEGs between the tumor and non-tumor tissues. To determine the survival significance of FRGs, we carried out univariate Cox proportional hazard regression analysis. The values of p < 0.05 were considered statistically significant. As a result, 37 DEGs were compliant with the criterion for further analysis. Using LASSO COX regression method [58], we selected the optimal FRGs from the high-dimensional data to build the signature. Finally, a prognostic formula for PCa was developed with 14 identified ferroptosis-related genes and their corresponding coefficients, which is as follows: risk score = ∑(coefficient_i _× expression of signature gene_i_). Specific information is shown in Supplementary Figure 2.

Based on the median risk score, the patients were classified as a high-risk group and a low-risk group. The performance of the FRG signature was evaluated by Kaplan-Meier analysis and the AUC value of the ROC curve.

### Function enrichment analysis for DEGs

To clarify the functions of differentially expressed FRGs, pathway enrichment analysis of the genes was conducted based on the kyoto encyclopedia of genes and genomes (KEGG) [59] and gene ontology (GO) databases, including the biological process (BP), molecular function (MF) and cellular component (CC) categories [60]. p<0.001 was considered statistically significant. The top 5 pathways were picked when the number of pathways was more than 5 for visual presentation by ggplot2 package.

### Gene set enrichment analysis

Knowing that GSEA is a computational algorithm for analyzing the molecular profiles of the data set [61], we compared the high- and low- risk group patients from TCGA dataset to identify enriched KEGG pathways, biological processes, cellular components, molecular functions, and dysregulated oncogenic characters related to the signature with reference to the C2 (curated gene sets), H(hallmark gene sets), C5 (GO gene sets), and C6 (Oncogenic signature) of Molecular Signatures Database (MSigDB) [62].

### Tumor immunity analysis

Knowing that CIBERSORT can estimate the enrichment degree of 22 different tumor-infiltrating immune cells (TIIC) using a deconvolution method [63], we utilized CIBERSORT and Spearman correlation to assess different distributions of immune cells with variation of the risk score. The Wilcoxon rank-sum test was carried out to examine the association between the signature and CD8+ T cell as well PD-L1.

### Detection of the mRNA and protein levels of key genes in clinical samples

Ten pairs of PCa and adjacent non-tumor tissues were obtained from Fudan University Huashan Hospital. Total RNA was extracted from the tissue samples, using RNAiso Plus reagent (Takara). Complementary DNA was synthesized from the extracted RNA using a cDNA reverse transcription kit (PrimeScript™ RT Master Mix, Takara). The mRNA expression level was quantitated by qRT-PCR using SYBR qPCR Master Mix (Vazyme). The relative expression of the target gene was calculated using the 2−ΔΔCt method (ΔCt = Cttarget gene-*in vitro* control). The primer sequences are shown in Supplementary Table 3. The Human Protein Atlas (HPA, version: 18.1) database (https://www.proteinatlas.org/) was applied to compare the protein expression of these genes in tumor and normal tissue [64].

### Statistical analysis

All statistical analyses were performed using RStudio and its appropriate packages. Differential expression analysis was executed with limma package. The “glmnet” package was used to conduct the Lasso Cox regression modeling. “survminer” package was employed for survival analysis. FDR method was utilized to adjust multiple testing. Multivariate Cox regression analysis was performed to adjust covariates for verifying independent risk factors of survival.

## Supplementary Material

Supplementary Figures

Supplementary Tables
