# Supplementary material for: The construction and analysis of a ferroptosis-related gene prognostic signature for pancreatic cancer
Source: Aging (Albany NY). 2021 Apr 4;13(7):10396–414. doi: 10.18632/aging.202801 (PMC8064155; doi:10.18632/aging.202801)
Supplement: Supplementary Figures [file aging-13-202801-s001.pdf]

SUPPLEMENTARY FIGURES

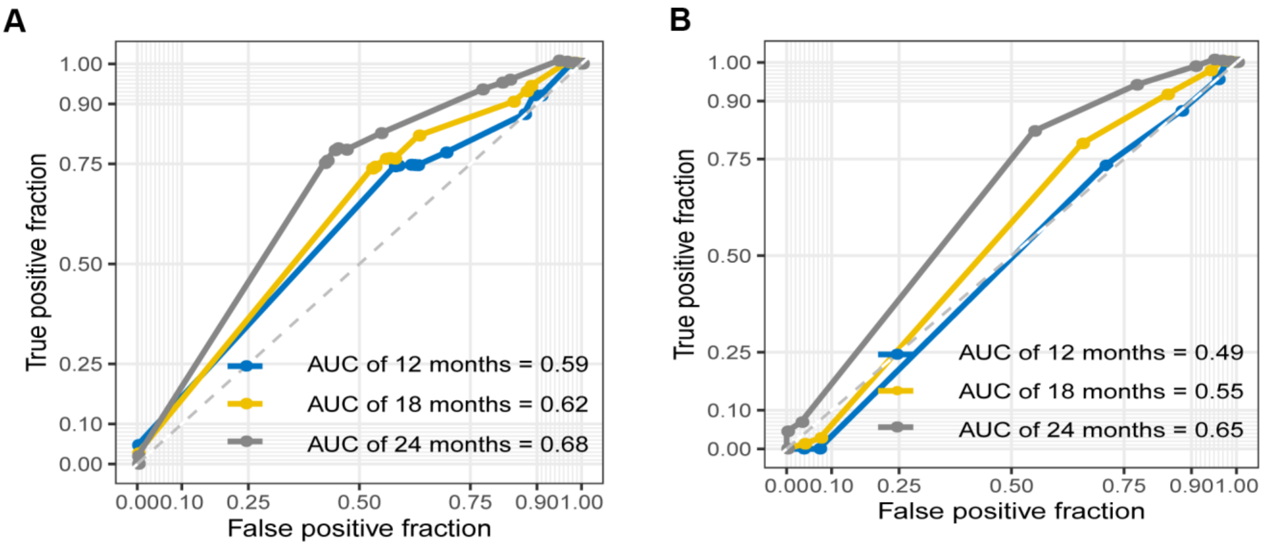

Supplementary Figure 1. Time-dependent ROC curves at 12, 18, 24 months for AJCC (**A**) and TNM (**B**).

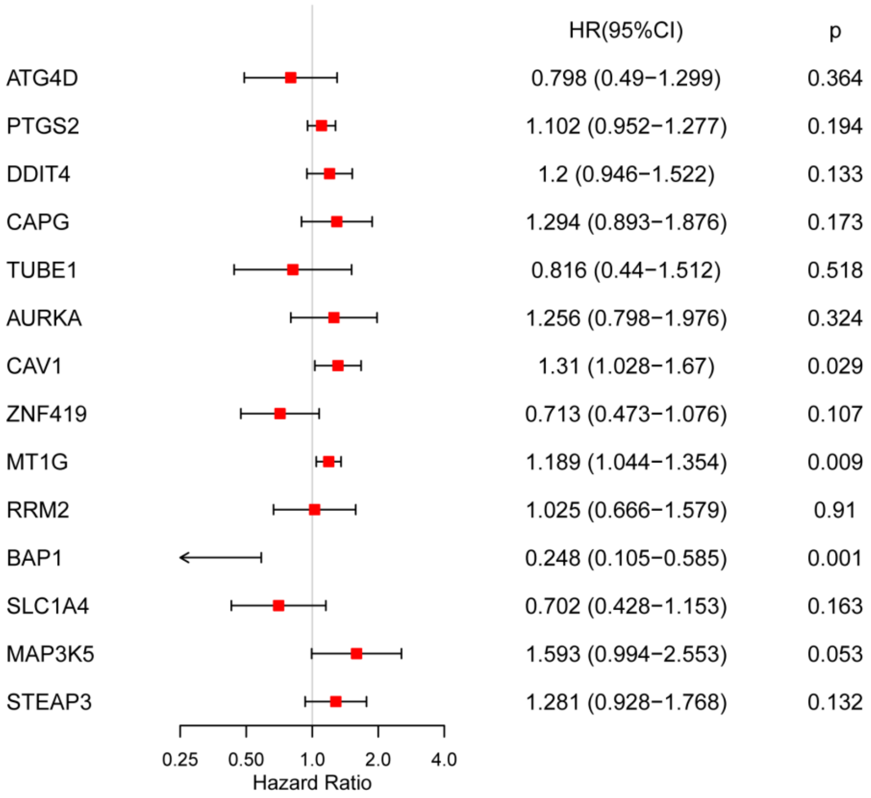

Supplementary Figure 2. Forest plot of GRGs associated with the survival of patients.
