# Supplementary material for: The construction and analysis of a ferroptosis-related gene prognostic signature for pancreatic cancer
Source: Aging (Albany NY). 2021 Apr 4;13(7):10396–414. doi: 10.18632/aging.202801 (PMC8064155; doi:10.18632/aging.202801)
Supplement: Supplementary Tables [file aging-13-202801-s002.pdf]

SUPPLEMENTARY TABLES

Supplementary Table 1. Patient characteristics in the TCGA-PAAD cohorts.

Variables	Number of cases	Percentage (%)
Age(year)		
≥60/<60	119/59	66.9/33.1
Gender		
Female/ Male	80/98	44.9/55.1
Tumor stage		
I-II/ III-IV	145/33	81.5/18.5
Grade		
G1-2/ G3-4	128/50	71.9/28.1
Chemotherapy		
YES/NO/NA	34/27/135	17.3/13.8/68.9
Radiotherapy		
YES/NO/NA	2/59/135	1.02/30.1/68.9
Drinking		
YES/NO/NA	105/68/23	53.6/34.7/11.7
Chronic pancreatitis		
YES/NO/NA	13/135/48	6.63/68.9/24.5
Diabetes		
YES/NO/NA	39/116/41	19.9/59.2/20.9
Family history of cancer		
YES/NO/NA	71/47/78	36.2/24/39.8

NA, not available.

Supplementary Table 2. Result of differential analysis to ARGs between tumor samples and non-tumor samples.

**Up-regulated genes**	DUOX2	TMBIM4	NQO1	PTGS2	GPX2	SLC2A1
	NOX4	MYB	NOX1	CAPG	TP63	CDKN2A
	SCD	NNMT	AURKA	NCF2	SLC2A12	HMOX1
	RRM2	CA9	STMN1	RGS4	SLC2A3	SLC7A5
	CYBB	ALOX15B	DPP4	PRDX1	CAV1	TLR4
**Down-regulated genes**	ALB	GLS2	BNIP3	PLIN4	PSAT1	GPT2
	MT1G	SLC2S14	XBP1	HBA1	HAMP	FLT3
	VLDLR	CXCL2	TUBE1	CBS	ULK1	NGB
	SLC1A4	TF	MAPK8	EGFR	HERPUD1	ATM
	ASNS	GABARAPL1		ARNTL	FBXW7	TAZ
	VDAC2	ATF4	ACO1	MIOX	DRD4	GDF15
	MAP3K5	JUN	SETD1B	CDO1	PRKAA2	MAPK9
	DDIT4	MT3	ATG4D	ALOX12	BRD4	NOX5
	ULK2	HSF1	CHAC1	LPCAT3	VEGFA	GABPB1
	PIK3CA	HNF4A	STEAP3	PCK2	FTH1	CS
	ACVR1B	BAP1	GCLC	WIPI1	ZNF419	CD44
	PEBP1	MTOR	NFS1			

Supplementary Table 3. The primers sequence of key ferroptosis related genes.

Gene	Forward primer	Reverse primer
ZNF419	TCCCCTCCAGCTCTACTCAC	CACATAGCCCTGCTGAGCG
TUBE1	CAGTGCGGAAACCAGATCG	AGAAGCTGCTTATTGCCTCATC
STEAP3	CTCCCCGGAGGTCATCTTTG	TCTTGCTCTGTAGGGTTGCTC
SLC1A4	TGTTTGCTCTGGTGTTAGGAGT	CGCCTCGTTGAGGGAATTGAA
RRM2	CACGGAGCCGAAAACTAAAGC	CGCCTCGTTGAGGGAATTGAA
PTGS2	CTGGCGCTCAGCCATACAG	CGCACTTATACTGGTCAAATCCC
MT1G	AAAGGGGCATCGGAGAAGTG	GCAAAGGGGTCAAGATTGTAGC
MAP3K5	CTGCATTTTGGGAAACTCGACT	AAGGTGGTAAAACAAGGACGG
DDIT4	TGAGGATGAACACTTGTGTGC	CCAACTGGCTAGGCATCAGC
CAPG	GGGGACTCCTACCTAGTGCTG	CACCACCTTCCTGGTACTTGA
CAV1	GCGACCCTAAACACCTCAAC	ATGCCGTCAAAACTGTGTGTC
BAP1	GCTCGTGGAAGATTTCGGTGT	TCATCAATCACGGACGTATCATC
AURKA	GAGGTCCAAAACGTGTTCTCG	ACAGGATGAGGTACACTGGTTG
ATG4D	GGAACAACGTCAAGTACGGTT	CTCGCCCTCGAAACGGTAG

Supplementary Table 4. Clinicopathological parameters of patients.

Case id	Age	Gender	Tumor size (cm)	Pathologic_T*	Pathologic_N*	Pathologic_M*	Overall survival (month)
1	61	female	2*1*1	T1	N0	M0	18.5
2	49	female	5*3*2.5	T3	N1	M0	12.5
3	63	male	3.5*3*3	T2	N1	M0	16
4	59	female	6*4.5*3	T3	N0	M0	17
5	52	female	4.5*3*2	T3	N0	M0	16
6	64	female	3.5*3*2	T2	N0	M0	7
7	69	male	2.5*2*2	T4	N0	M0	8.5
8	49	male	5*3*2	T4	N1	M0	0
9	77	female	3*2*1.5	T2	N1	M0	3
10	56	male	3.5*3*2	T2	N0	M0	17.5

Supplementary Table 4. Clinicopathological parameters of patients (Continued).

Case id	Chemotherapy	Radiotherapy	Drinking	Chronic pancreatitis	Diabetes	Family history of cancer
1	1	0	0	0	0	0
2	1	0	0	0	0	0
3	1	0	0	0	1	0
4	1	0	0	0	0	0
5	1	1	0	0	1	0
6	1	0	0	0	0	0
7	1	0	0	0	0	0
8	0	0	0	0	0	0
9	1	0	0	0	0	0
10	1	0	0	0	0	0

* tumor node metastasis.
